# Migraine abortive treatment in children and adolescents in Israel

**DOI:** 10.1038/s41598-022-11467-3

**Published:** 2022-05-06

**Authors:** Jacob Genizi, Dana Lahoud, Rony Cohen

**Affiliations:** 1 Pediatric Neurology Unit, Bnai Zion Medical Center, Haifa, Israel; 2 Pediatric Department, Bnai Zion Medical Center, Haifa, Israel; 3 Bruce and Ruth Rappaport Faculty of Medicine, Technion, Haifa, Israel; 4 Department of Pediatric Neurology, Schneider Children’s Medical Center of Israel, Petah Tikva, Israel

**Keywords:** Medical research, Neurology

## Abstract

Migraine headaches in children may cause attacks that require abortive treatment. This study evaluated the incidence and efficacy of medications used for relieving migraine headache attacks in the pediatric population in Israel. Children 6–18 years of age who were diagnosed in our pediatric neurology clinic as having migraine headaches were enrolled into the study. Children and their parents recorded the children response to abortive treatment during consecutive migraine attacks. Fifty children, with 116 migraine attacks, were included in the study (30 females; mean age 12; range 6–18). Forty-seven (94%) reported on abortive treatment on the first migraine attack, 43 (86%) on a second migraine attack and 26 (52%) on a third migraine attack. During the first recorded migraine attack, 41 children (87.5%) reported taking only one type of medication for each headache episode, mainly ibuprofen or acetaminophen; less than a quarter used dipyrone (metamizol). Overall the improvement rate after two hours was 65.4% ± 27 for ibuprofen, 59.8 ± 35.3 for acetaminophen and 50.9 ± 27.4 for dipyrone without statistical difference. However, in the first recorded headache episode, males had a significantly better response to acetaminophen, compared to ibuprofen (95% ± 28 vs 75 ± 20). In conclusion, Children with migraine in Israel mainly use a single medication for each headache episode. Ibuprofen is the most commonly used abortive treatment; however, acetaminophen was associated with a better response among some of our patients.

## Introduction

Headaches are a common complaint in children and adolescents^1^, caused in most cases by a benign illness or a primary headache disorder. Migraine and tension-type headache are the two most common headache disorders among children, and are distinguished clinically by their characteristics and accompanying features according to the IHS criteria^2^. Frequent migraine headache attacks can cause a significant impact on functionality and quality of life, prompting the need for early recognition and treatment^3–6^. Thus, the goal of acute treatment of migraine headaches should be a rapid return to normal function with minimal side effects. Patients should be instructed to treat the headache as quickly as possible^7^. Along with treating the acute headache attack, support therapies should be offered by the pediatric neurologist, pediatrician, psychologist, and support groups to reduce the frequency of attacks^8^. An interdisciplinary treatment (including pediatric neurology, psychology, physiotherapy and others) which is based on the bio-psycho-social model of pain is recommended in children with migraine.

Ibuprofen and acetaminophen are the analgesics most commonly used for migraine in children, and their safety has been verified in controlled trials^9^. Dipyrone (metamizol) is also used in many countries, including Israel, but is not available in others, particularly the USA and UK, because of its association with potentially life‐threatening blood dyscrasias (agranulocytosis)^10^. On the other hand, triptans that are widely used for migraine acute treatment are not recommended for children in Israel. Several studies have examined the efficacy of acetaminophen and ibuprofen for the acute treatment of migraine in children. In a double-blind crossover study of 88 children, both drugs were shown to be effective treatments for severe to moderate migraine attacks in children, with ibuprofen giving the best relief^11^. A similar study comparing ibuprofen and placebo in a group of younger children also found benefits for ibuprofen, although with a slight male-to-female difference^12^.

The efficacy of dipyrone for the acute treatment of primary headaches in children has not been studied in well-controlled trials. Evidence from a small number of trials suggests that dipyrone is effective for episodic tension-type headache and migraine. No serious adverse events were observed in the included trials, but agranulocytosis is rare and would probably not be observed in the relatively small sample examined^13^.

In addition, as yet, no studies have been conducted to compare the treatments commonly used in Israel for acute migraine headache attacks among children. Our goal was to evaluate and compare the efficacy of the medications used for relieving migraine headache attacks in the pediatric population in Israel.

## Results

Of the 50 patients who initially entered the study, 47 (94%) reported using abortive treatment on the first migraine attack (30 females; mean age 12; range 6–18). Forty-three patients (86%) reported on a second migraine attack (median 44.5 days from the first report; range 0–297; 23 females, median age 12), and 26 (52%) reported on a third migraine attack (median 55 days from the first report; range 3–265; 15 females, median age 12). See Table 1.

**Table 1:** Abortive migraine treatment reported in the three headache attacks.

	Total	Acetaminophen only N (%)	Ibuprofen only N (%)	Dipyrone only N (%)	More than one medication N (%)
First headache attack	47	15 (31.9%)	19 (40.4%)	7 (14.9%)	6 (12.8%)
Second headache attack	43	11 (25.6%)	22 (51.2%)	6 (14.05)	4 (9.3%)
Third headache attack	26	6 (23.1%)	14 (53.8%)	3 (11.5%)	3 (11.5%)

Headache characteristics in the sample were as follows: on average, the headaches began about two years prior to patients filling out the first headache attack (mean 24 months, range 1–96 months). Of the 50 children, 26% (13) experienced aura. The most common location of the headache was the frontal region of the head (28; 65%), followed by the temporal region of the head (12; 28%) and behind the eyes (4; 9%). More than two thirds (34; 68%) reported bilateral headaches. Thirty-four children (68%) described their headaches as pressing, 13 (26%) as throbbing, and 2 (4%) as both pressing and throbbing. Headaches were largely rated as severe (median 8/10; range 4–10) and lasted for a median of 5 h (mean 12.2 h; range 2–90 h). The children had a median of 5.5 headache episodes per month (mean 7.8, range 1–14). Eleven children (22%) had mixed headaches (i.e. reported also having headaches that met the criteria for episodic TTH). Thirty-one (62%) had photo sensitivity and 22 (44%) phono sensitivity. In 24 children (48%) the headaches were accompanied by nausea and in 18 (36%) by vomiting. Most (39; 78%) obtained some relief by lying down, and 70% (35) reported substantial relief only from medication.

During the first headache attack, as noted above, 47 of 50 patients (94%) reported taking abortive treatment for headache episodes. Of these, 41 (87.5%) reported taking only one type of medication for each headache episode, with 22 (44%) using ibuprofen (10 mg/kg, maximum 400 mg), 20 (40%) using acetaminophen (15 mg/kg; maximum 500 mg), and 11 (22%) using dipyrone (15 mg/kg; maximum 500 mg). The remaining six children (12.8%) used more than one medication (The medications were not taken together but an hour after the failure of the first medication): three (6%) used both dipyrone and acetaminophen, two (4%) used both ibuprofen and acetaminophen, and one child (2%) used both dipyrone and ibuprofen.

Table 2 presents demographic and headache characteristics by pain relief treatment based on the first headache attack. There was a statistically significant difference in age with respect to the pain relief used: children who used ibuprofen were significantly younger than those who used dipyrone (mean difference = − 3.73, p < 0.03). No other significant demographic or headache characteristics were found.

**Table 2:** Demographic and headache characteristics by pain relief treatment. Data are given as mean ± SD (median, range) or N (%).

	Ibuprofen (N = 22)	Acetaminophen (N = 20)	Dipyrone (N = 11)	χ^2^	P
Age (years)	11.3 ± 3.2 (11.0; 7–17)	12.3 ± 3.0 (12.5; 6–17)	14.6 ± 2.4 (15.0; 10–18)	3.96*	0.03
Start of headaches (months)	22.9 ± 27.6 (12; 1–96)	20.4 ± 22.6 (12; 1–84)	34.0 ± 35.1 (15.0; 3–96)	1.17	0.56
**Sex**				0.54	0.76
Male	9 (40.9%)	6 (30.0)	4 (36.4)		
Female	13 (59.1%)	14 (70.0)	7 (63.6)		
Maximum pain level	8.0 ± 1.4 (8; 5–10)	7.5 ± 1.6 (8; 5–10)	8.0 ± 1.7 (9; 4–10)	2.21	0.33
Duration of pain (h)	16.4 ± 21.0 (9.5; 1–90)	10.5 ± 14.4 (3.5; 1–48)	12.8 ± 15.2 (5; 1–48)	1.70	0.43
Number of events per month	6.9 ± 7.8 (4.0; 1–30)	10.0 ± 8.6 (7.5; 1–30)	8.6 ± 8.1 (7.0; 1–30)	3.99	0.14
Bilateral	14 (63.6)	16 (80.0)	6 (54.5)	2.43	0.30
**Location of headache**					
Frontal	8 (36.4)	13 (65.0)	7 (63.6)	4.09	0.13
Temporal	7 (31.8)	3 (15.0)	2 (18.2)	1.84	0.45
Eye	3 (13.6)	0 (0.0)	1 (9.1)	2.84	0.25
**Type of pain**				8.05	0.09
Pressing	13 (59.1)	16 (84.2)	8 (72.7)		
Throbbing	8 (36.4)	3 (15.8)	1 (9.1)		
Both	1 (4.5)	0 (0.0)	2 (18.2)		
Nausea	11 (50.0)	10 (50.0)	6 (54.5)	0.07	0.97
Vomiting	11 (50.0)	5 (25.0)	2 (18.2)	4.46	0.11
Photo sensitivity	13 (59.1)	10 (50.0)	8 (72.7)	1.52	0.47
Phono sensitivity	11 (50.0)	8 (40.0)	5 (45.5)	0.42	0.81
Aura	7 (31.8)	3 (15.0)	3 (27.3)	1.65	0.50
**Eased by**					
Lying down	19 (86.4)	16 (80.0)	6 (54.5)	4.37	0.11
Medication	17 (77.3)	13 (65.0)	8 (72.7)	0.79	0.68
Darkness	10 (45.5)	7 (35.0)	5 (45.5)	0.56	0.76

*F.

There were also no significant differences in demographic or headache characteristics based on the number of medications taken (one vs. more than one), except that children who took more than one type of medication during a given headache episode had more difficulty falling asleep.

Forty three children reported on the second headache attack (time from previous report: median 44.5 days, range 1–297; 23 females, median age 12). Of these, 39 (90.0%) reported taking only one type of medication for each headache episode, with 22 (51.2%) using ibuprofen (10 mg/kg; maximum 400 mg), 11 (25.6%) acetaminophen (15 mg/kg; maximum 500 mg), and 6 (14.05%) dipyrone (15 mg/kg; maximum 500 mg). The other four children (9.3%) used more than one medication: ibuprofen and acetaminophen (two children); ibuprofen and dipyrone (one child), or acetaminophen and dipyrone (one child).

Forty patients provided data on abortive medication in two consecutive reports. Of these, 30 (75%) used the same medication for both episodes, and 10 (25%) switched to a different medication. Six switched to ibuprofen (five from acetaminophen and one from dipyrone), three to acetaminophen (two from ibuprofen and one from dipyrone), and one to dipyrone (from acetaminophen). Children who switched medication reported higher pain levels and more frequent waking from sleep in their first report compared to children who did not switch their medication. In addition, children who switched medication reported fewer monthly events at the first report. No other headache measures at the second report were associated with switching medication, except that those who switched were more likely than those who did not, to report that darkness eased their pain.

Twenty three children reported on the third headache attack (time from previous report: median 55 days, range 3–265; 15 females, median age 12). Of these, 23 (88.5%) reported taking only one type of medication for each headache episode, with 14 (53.8%) taking ibuprofen (10 mg/kg; maximum 400 mg), 6 (23.1%) taking acetaminophen (15 mg/kg; maximum 500 mg), and 3 (11.5%) taking dipyrone (15 mg/kg; maximum 500 mg). The remaining three (11.5%) used more than one medication.

Also, at the third headache attack, 19 (73%) children used the same medication as in their first report, and 7 (27%) switched to a different medication. Twenty-two (84%) used the same medication as in their second report.

Children and parents separately graded the child’s response to treatment (in percentage terms) two hours after use. There was no statistically significantly difference between the assessments of the children and their parents with respect to any of the medications. Table 3 presents the children’s and parents’ evaluations of the treatment response based on data from all three headache attacks.

**Table 3:** Response to treatment. Data are given as mean ± SD (median; range).

	Child (% improvement)	Parent (% improvement)	Paired t	P
Ibuprofen (N = 55)	65.4% ± 27 (70; 10–100)	67.3 ± 26.6 (75; 10–100)	− 2.01	0.06
Acetaminophen (N = 32)	59.8 ± 35.3 (75; 0–100)	60.0 ± 35.8 (80; 0–100)	− 0.27	0.79
Dipyrone (N = 16)	50.9 ± 27.4 (50; 10–90)	56.4 ± 26.6 (50; 10–100)	− 1.75	0.11

Table 4 displays the children’s and parents’ evaluations for the three medications by gender for the three headache attacks separately. As can be seen, in the first headache attack, among male gender, acetaminophen was associated with a better response (P = 0.001). In general, male gender was associated with a better response than female gender (particularly for acetaminophen in the first headache attack and ibuprofen in the third). The duration of the headache attacks was negatively associated with the patient’s evaluation of ibuprofen; children who had shorter headache attacks, had better response to ibuprofen. No other headache characteristics were associated with ibuprofen usage. No associations were found for evaluations of dipyrone.

**Table 4:** Comparison of patients’ and parents’ evaluation of treatment response for each medication by gender and headache attack.

	Ibuprofen (%)		Acetaminophen (%)		Dipyrone (%)	
	Child	Parent	Child	Parent	Child	Parent
**First headache attack**						
Male	75.0 ± 23.0	75.0 ± 20.0	95.0 ± 28.0	95.0 ± 28.0	45.0 ± 18.0	55.0 ± 25.0
Female	50.0 ± 25.0	60.0 ± 25.0	62.5 ± 20.0	55.0 ± 20.0	50.0 ± 10.0	50.0 ± 10.0
p	0.28	0.38	0.009	0.01	0.65	0.79
**Second headache attack**						
Male	76.5 ± 25.8	79.0 ± 28.1	60.0 ± 34.2	60.0 ± 33.4	65.0 ± 19.1	67.5 ± 18.9
Female	68.8 ± 26.0	71.6 ± 24.8	63.3 ± 33.9	65.8 ± 34.7	72.5 ± 26.3	72.5 ± 26.3
p	0.47	0.48	0.86	0.76	0.66	0.77
**Third headache attack**						
Male	83.8 ± 19.2	86.2 ± 15.0	70.0 ± 42.4	65.0 ± 49.5	80.0	80.0
Female	40.0 ± 36.6	42.5 ± 37.3	52.1 ± 35.3	56.4 ± 34.0	86.7 ± 11.5	83.3 ± 26.3
p	0.01	0.01	0.56	0.78	0.67	0.87

## Discussion

In this prospective cohort study of migraine abortive treatment in Israeli children, we found that acetaminophen and ibuprofen were the most commonly used drugs, with a slight preference for ibuprofen (44% vs. 40%). The choice between acetaminophen and ibuprofen was not influenced by the patient’s gender or age. Although dipyrone is registered in Israel, only about 20% of the patients used it, with slightly more of these being older children.

The aim of migraine headache treatment is to achieve fast, complete pain relief. Early treatment of migraine in adults has been shown to increase the amount of time patients spend pain-free^7^. In the pediatric population as well, early treatment is likely to improve efficacy. In particular, it is increasingly recognized that many children and adolescents benefit from nonprescription oral analgesics like acetaminophen or ibuprofen^14,15^. Triptans are less commonly prescribed in children because of registry problems^14^. In our study as well, triptans were not used to abort any headache attacks.

In our study, most of the children used only one drug during each headache attack, however they reported a fair response (between 50 and 95%). Our findings are in line with other studies; Richer et al.^16^ and Hamalainen et al.^17^.

Richer et al.^16^, in a Cochrane Review, reported that triptans, ibuprofen and acetaminophen were all found to be effective in treating migraine headaches. However, he reported a response rate of 62% for zolmitriptan, and 69% for ibuprofen. Hamalainen et al.^17^ reported the rate of 60% for ibuprofen and 39% for acetaminophen.

It should be noted that we allowed patients to add another abortive medication if the first one failed, but in each headache attack only 9–12.8% of our patients did so. To our knowledge, our study is the first to report on multiple medication use.

Our study is also unique in our follow-up. We asked our patients to report during three different headache episodes, in order to examine the consistency of patients’ preference in their selection of medications. Most of our patients (73%) used the same medication during all three headache episodes, further supporting the good response to over-the-counter medication in pediatric migraine. Those who switched medications between headache episodes graded their pain as more intense, although overall they had fewer episodes. It appears that, in children, abortive treatment is chosen in response to a specific episode and is less influenced by the overall frequency of the headache attacks.

While we did not compare ibuprofen and acetaminophen head to head, we note that our patients reported a better response to acetaminophen. Hamalainen et al.^17^, in an old study, reported better headache relief in migrainous children using ibuprofen.

Damen et al.^18^, in a systematic review, found both acetaminophen and ibuprofen to be effective in reducing migraine headache symptoms, with no clear difference between them. In a Cochrane Review^16^ very few randomized control studies were found on migraine abortive treatment, and most focused on triptans. Ibuprofen was found to be more effective than placebo for producing pain freedom, but with low-quality evidence.

In our study about 20% of the children used dipyrone, and reports on pain relief from dipyrone (50.9 ± 27.4) were slightly lower but not significantly different from those for acetaminophen and ibuprofen. Tulunay et al.^19^, in a double-blind study, found a significant improvement in pain with dipyrone compared to placebo. However, Leeuw et al.^20^ found no evidence to support the claim that dipyrone is superior or even equivalent to ibuprofen in treating pediatric pain. Ramacciotti et al.^13^, in a Cochrane Review, reported that a small number of trials suggest that dipyrone is effective for migraine. No serious adverse events were observed in the included trials, but agranulocytosis is rare and would probably not be observed in such a relatively small sample. None of those studies were conducted in children.

In our study, boys responded better than girls. Lewis et al.^21^ also found that boys responded better to ibuprofen compared to girls. Lewis suggested that younger girls (6–12 years) have an innate or socially driven desire to please, thereby yielding a higher placebo response rate. Our study included older children, with a mean age of 12 years. The better response rate in boys may also relate to a difference between the genders in pain complaints. Girls were found to have more somatic complaints^22^, and their response to abortive treatment may also be reduced, although other pain studies have failed to demonstrate this.

Our study has some limitations. It is based on a single clinic, with a relatively small number of patients. Our setting is unique in that the mainstay pharmacological regimen differs from other countries. Specifically, dipyrone is prescribed whereas the use of triptans is not recommended for children. Accordingly, our results are not generalizable to all countries.

## Conclusions

Children with migraine in Israel mainly use a single medication for each headache episode. Ibuprofen is the most commonly used abortive treatment; however, among some patients, acetaminophen was associated with a better response. Dipyrone was used by only a small number of patients, and was not superior to the other medications used. Larger studies are needed to support our findings.

## Patients and methods

The study group comprised 50 children 6–18 years of age (30 females [60%]; mean age 12 years) who were assessed in the pediatric neurology clinic of a large hospital in northern Israel between September 2019 and June 2021, and who were diagnosed as having migraine headache according to ICHD 3^2^. All methods were carried out in accordance with relevant guidelines and regulations. All experimental protocols were approved by the Bnai Zion institutional Helsinki committee number BNZ 0095-19. Informed consent was obtained from the parents of all children involved in the study. The children and their parents were asked to report (using a paper diary) about their use of analgesics (dipyrone, ibuprofen, acetaminophen, or any combination of these) during three consecutive acute headache attacks, after entering the study. Response two hours after abortive use was rated separately by the patient and his/her parents, in percentage terms (e.g., 60% improvement).

Statistical methods were as follows: differences in demographic and headache characteristics between patients using different types of medication were assessed by chi-square test for categorical data and the Kruskal–Wallis test or one-way ANOVA (for age) for continuous data. Differences in demographic and headache characteristics between patients using different numbers of medications were assessed by chi-square test for categorical data and the independent t-test or the Mann–Whitney test where appropriate for continuous data. The Wilcoxon signed rank test was used to compare medication dose between the first and second headache attack. Paired t-tests were used to test differences between parents’ and children’s assessments of the response to treatment. Spearman’s correlation was used to assess associations between reaction to treatment (parent/child) and the demographic data, and between reaction to treatment and headache characteristics.

## References

[1] Genizi J, Srugo I, Kerem NC (2016). Primary headache in children and adolescents: From pathophysiology to diagnosis and treatment. J. Headache Pain Manag..

[2] Headache Classification Committee of the International Headache Society (IHS) (2018). The international classification of headache disorders, 3rd edition. Cephalalgia.

[3] Malone CD, Bhowmick A, Wachholtz AB (2015). Migraine: Treatments, comorbidities, and quality of life, in the USA. J. Pain Res..

[4] Smith TR, Nicholson RA, Banks JW (2010). Migraine education improves quality of life in a primary care setting. Headache.

[5] Powers S, Patton S, Hommel K, Hershey A (2003). Quality of life in childhood migraines: Clinical impact and comparison to other chronic illnesses. Pediatrics.

[6] Hershey AD, Winner PK (2005). Pediatric migraine: Recognition and treatment. Am. Osteopath. Assoc..

[7] Lanteri-Minet M, Mick G, Allaf B (2012). Early dosing and efficacy of triptans in acute migraine treatment: The TEMPO study. Cephalalgia.

[8] Genizi J (2017). Paediatric primary headache: Pharmacological and non-pharmacological treatments. Eur. Med. J. Neurol..

[9] Lewis DW, Winner P (2006). The pharmacological treatment options for pediatric migraine: An evidence-based appraisal. NeuroRx.

[10] Hedenmalm K, Spigset O (2002). Agranulocytosis and other blood dyscrasias associated with dipyrone (metamizole). Eur. J. Clin. Pharmacol..

[11] Hamalainen ML, Hoppu K, Valkeila E, Santavuori P (1997). Ibuprofen or acetaminophen for the acute treatment of migraine in children. Neurology.

[12] Lewis DW, Kellstein D, Dahl G, Burke B, Frank LM, Toor S, Northam RS, White LW, Lawson L (2002). Children's ibuprofen suspension for the acute treatment of pediatric migraine. Headache.

[13] Ramacciotti AS, Soares BG, Atallah AN (2014). Dipyrone for acute primary headaches. Cochrane Database Syst. Rev..

[14] Oskoui M, Pringsheim T, Holler-Managan Y (2019). Practice guideline update summary: Acute treatment of migraine in children and adolescents. Report of the Guideline Development, Dissemination, and Implementation Subcommittee of the American Academy of Neurology and the American Headache Society. Neurology.

[15] Bigal ME, Lipton RB, Winner P (2007). Migraine in adolescents: Association with socioeconomic status and family history. Neurology.

[16] Richer L, Billinghurst L, Linsdell MA (2016). Drugs for the acute treatment of migraine in children and adolescents. Cochrane Database Syst. Rev..

[17] Hämäläinen ML, Hoppu K, Valkeila E, Santavuori P (1997). Ibuprofen or acetaminophen for the acute treatment of migraine in children: A double-blind, randomized, placebo-controlled, crossover study. Neurology.

[18] Damen L, Bruijn JK, Verhagen AP, Berger MY, Passchier J, Koes BW (2005). Symptomatic treatment of migraine in children: A systematic review of medication trials. Pediatrics.

[19] Tulunay FC, Ergun H, Gulmez SE (2004). The efficacy and safety of dipyrone (Novalgin) tablets in the treatment of acute migraine attacks: A double-blind, cross-over, randomized, placebo-controlled, multi-center study. Funct. Neurol..

[20] de Leeuw TG, Dirckx M, Gonzalez Candel A, Scoones GP, Huygen FJPM, de Wildt SN (2017). The use of dipyrone (metamizol) as an analgesic in children: What is the evidence? A review. Paediatr. Anaesth..

[21] Lewis DW, Kellstein D, Dahl G (2002). Children’s ibuprofen suspension for the acute treatment of pediatric migraine. Headache.

[22] Genizi J, Srugo I, Kerem NC (2013). The cross-ethnic variations in the prevalence of headache and other somatic complaints among adolescents in Northern Israel. J. Headache Pain.

